# Novel Presentation of Major Histocompatibility Complex Class II Deficiency with Hemophagocytic Lymphohistiocytosis

**DOI:** 10.1007/s10875-024-01674-0

**Published:** 2024-03-01

**Authors:** Fayhan J. Alroqi, Musaab A. Alhezam, Abdullah I. Almojali, Tlili Barhoumi, Nouf Althubaiti, Yousef Alharbi, Mohammed A. Al Balwi, Abdulrahman Alrasheed

**Affiliations:** 1 Division of Pediatric Allergy and Immunology, Department of Pediatrics, King Abdullah Specialized Children’s Hospital, King Abdulaziz Medical City, Riyadh, 14611 Saudi Arabia; 2 King Abdullah International Medical Research Center, Ministry of National Guard-Health Affairs, Riyadh, 14611 Saudi Arabia; 3 College of Medicine, King Saud bin Abdulaziz University for Health Sciences, Riyadh, 14611 Saudi Arabia; 4 Division of Pediatric Rheumatology, Department of Pediatrics, King Abdullah Specialized Children’s Hospital, King Abdulaziz Medical City, Riyadh, 14611 Saudi Arabia; 5 Pathology and Laboratory Medicine Department, King Abdulaziz Medical City, Ministry of National Guard Health Affairs, Riyadh, 14611 Saudi Arabia

**Keywords:** MHC-II deficiency, HLH, Tuberculosis, RFXANK, Hyperinflammation

## Abstract

**Purpose:**

Major histocompatibility complex (MHC) class II deficiency is one of the combined immune deficiency disorders caused by defects in the MHC class II regulatory genes leading to abnormal T cells development and function. Therefore, patients mainly present with increased susceptibility to infections, diarrhea, and failure to thrive. In this report, we present one MHC class II deficient patient with a novel presentation with Hemophagocytic Lymphohistiocytosis (HLH).

**Methods:**

Immunophenotyping of lymphocyte subpopulations and HLA-DR expression was assess by flow cytometry. Gene mutational analysis was performed by whole exome and Sanger sequencing.

**Results:**

We reported a 7-year-old girl, who was diagnosed at age of 2 years with MHC class II deficiency by genetic testing and flow cytometry. Two years later, she developed disseminated BCGitis which was treated with proper antimicrobial agents. At the age of 7 years, she presented with clinical features fulfilling 6 diagnostic criteria of HLH including evidence of hemophagocytic activity in bone marrow aspiration. Accordingly, the diagnosis of HLH was established and the patient was started on IV Dexamethasone, Anakinra and IVIG. Eventually, patient started to improve and was discharged in good condition. Few months later, the patient was readmitted with severe pneumonia and sepsis leading to death.

**Conclusion:**

Patients with MHC class II deficiency might present with disseminated BCGitis especially if the patient has severe T cell lymphopenia. Additionally, this immune defect might be added to the list of inborn errors of immunity that can be complicated with HLH.

**Supplementary Information:**

The online version contains supplementary material available at 10.1007/s10875-024-01674-0.

## Introduction

Major histocompatibility complex (MHC) class II deficiency is one of the autosomal recessive primary immune deficiency disorders that leads to combined immunodeficiency [1]. This mode of inheritance explains the increased frequency of this disease in consanguineous populations like the Middle East [2, 3].

This disorder is due to defects in the MHC class II regulatory genes that encode for CIITA, RFXANK, RFX5, and RFXAP. These proteins are required for the expression of MHC II molecule on the surface of antigen presenting cells (dendritic cells, B-cells and monocyte/macrophage lineage cells). This molecule is also expressed in other type of cells after activation by interferon gamma (IFN- γ) or other inflammatory signals [2, 4, 5].

Defects in MHC class II protein affect T cell development and function as this protein accounts for the development, activation, and homeostasis of T-helper (T_H_) cells. This ultimately will affect the B-cells function which explains the impaired cellular and humoral immunity in patients with MHC class II deficiency [6].

Therefore, MHC class II deficient patients are prone to severe viral, bacterial, fungal, and protozoal infections that might occur early in life [4, 6]. Accordingly, they might present with recurrent sino-pulmonary infections, oral thrush, and protracted diarrhea with severe malabsorption leading to failure to thrive. Interestingly, a huge number of MHC class II deficient patients had received Bacille Calmette-Guerin (BCG) vaccine as part of the national vaccination program in many countries, the majority of these patients did not develop disseminated infection [3, 4]. In fact, disseminated BCGitis was reported in only two MHC class II deficient patients [7, 8]. The rare occurrence of mycobacterial infections in patients with MHC calls II deficiency is not well understood.

Despite knowing the fact that this monogenic disorder usually leads to an immunodeficiency phenotype, few patients were reported to have autoimmune and hyperinflammatory features. A study of 35 MHC class II deficient patients with a same founder *RFXANK* mutation showed that two patients had autoimmune cytopenia [4]. Another retrospective study of 25 patients described two siblings with *CIITA* mutation who had hyperinflammatory and autoimmune phenomena. The first sibling was initially diagnosed with polyarticular juvenile idiopathic arthritis (JIA) at 14 months of age, which was resistant to multiple biological agents. At the age of 7 years, she was diagnosed with MHC class II deficiency following an immunological evaluation of prolonged fever. Her younger sister was investigated, as she had chronic diarrhea since birth, and found to have the same mutation. According to the report, both siblings developed picture of macrophage activation syndrome (MAS)/HLH with fever, cytopenia, high ferritin, elevated triglycerides and low fibrinogen prior to bone marrow transplantation (BMT). Both patients were managed with steroid and cyclosporine with good response [8].

HLH is a life-threatening condition due to uncontrolled hyperinflammation and organ infiltrations by multiple immune cells including Histiocytes, Cytotoxic T (CTLs), and Natural Killer (NK) cells. The clinical criteria for this disorder include the following: prolonged fever, splenomegaly, pancytopenia, hyperferritinemia, hypertriglyceridemia, hypofibrinogenemia, low or absent NK-cell activity, elevated soluble interleukin-2 receptor, and hemophagocytosis in bone marrow, spleen, or lymph nodes [9]. This disorder is usually classified as either primary monogenic or secondary disorder. Primary HLH is due to defects in CTL and NK cytotoxicity, caused by genetic defects in perforin or molecules required for lytic granule exocytosis. On the other hand, secondary HLH is a complication of a variety of conditions including malignancy and inborn error of immunity like chronic granulomatous disease, severe combined immunodeficiency disease and others [10–14].

In this report, we present one MHC class II deficient patient with a novel presentation with hyperinflammation and HLH. In addition, this patient will be the third reported case of MHC class II deficiency who had disseminated BCGitis.

## Materials and Methods

### Patient

The patient’s history, clinical presentation, and laboratory investigations are presented in the Results section. Sample collections were obtained from patient during her visit and follow up at immunology clinic for medical evaluation and a written informed consent was signed by the patient’s parents according to King Abdulaziz Medical City (KAMC) protocol following the Saudi Ministry of Health (MOH) guidelines.

### Antibodies and Flow Cytometry

Anti-human monoclonal antibodies (mAbs) to the following antigens were used for staining: CD3 (UCHT1), CD4 (RPA-T4), CD8 (SKI), CD14 (61D3), HLA-DR (LN3), CD19 (SJ25C1), CD25 (2A3), IFN-γ (4 S.B3), TNF-α (MAb11) and the appropriate isotype controls. Intracellular cytokine flow cytometry was obtained after 4-h stimulation with PMA/ionomycin.

### Genetic Studies

#### Molecular Genetic Analysis

Genomic DNA was extracted from patient’s whole blood using QIAamp DNA Mini Kit (Qiagen, Germany) according to the manufacturer’s instructions. Extracted DNA was then subjected to whole exon sequencing (WES) and Sanger sequencing.

#### Whole Exome Sequencing (WES)

WES was performed on genomic DNA focusing on genes that associated with primary immunodeficiency disorders and/or HLH (Supplementary data) using Illumina platform with an average coverage depth of ~ 100× where the Agilent SureSelect Human all exon enrichment kit (Santa Clara, CA) was used according to the manufacturer’s protocol. The library was sequenced using an Illumina HiSeq2000 instrument. Base calling, alignment of reads to GRCh37/hg19 genome assembly, primary filtering out of low-quality reads and probable artifacts, and subsequent annotation of variants was performed using in-house bioinformatics pipeline. Typically, ~ 97% of the targeted bases are covered > 10×. All disease-causing variants were checked against HGMD (http://www.hgmd.cf.ac.uk/ac/all.php), ClinVar (dSNPs; http://www.ncbi.nih.gov), Genome Aggregation Database (*gnomAD*) (https://gnomad.broadinstitute.org) and Exome Aggregation Consortium (*ExAC*) (http://exac.broadinstitute.org). Furthermore, the in-silico tools SIFT (http://sift.jcvi.org/), PolyPhen-2 (http://genetics.bwh.harvard.edu/pph2/), and MutationTaster (http://www.mutationtaster.org) were used to predict coding variant effects on protein function.

#### Sanger Sequencing Analysis

The identified homozygous *RFXANK* variant was confirmed by sanger sequencing using specific designed bidirectional primers, amplified by PCR and ABI prism Big Dye Terminator v. 3.1 cycle sequencing kit (Applied Biosystems, Foster City, CA) in Genetic Analyzer 3500 instrument (Thermofisher Scientific). Mutations were identified by comparison to reference sequence (NM_003721.3; http://www.ncbi.nlm.nih.gov) using Mutation Surveyor DNA Variant Analysis software v 5.0 (SoftGenetics, USA).

## Result

**Fig. 1:**
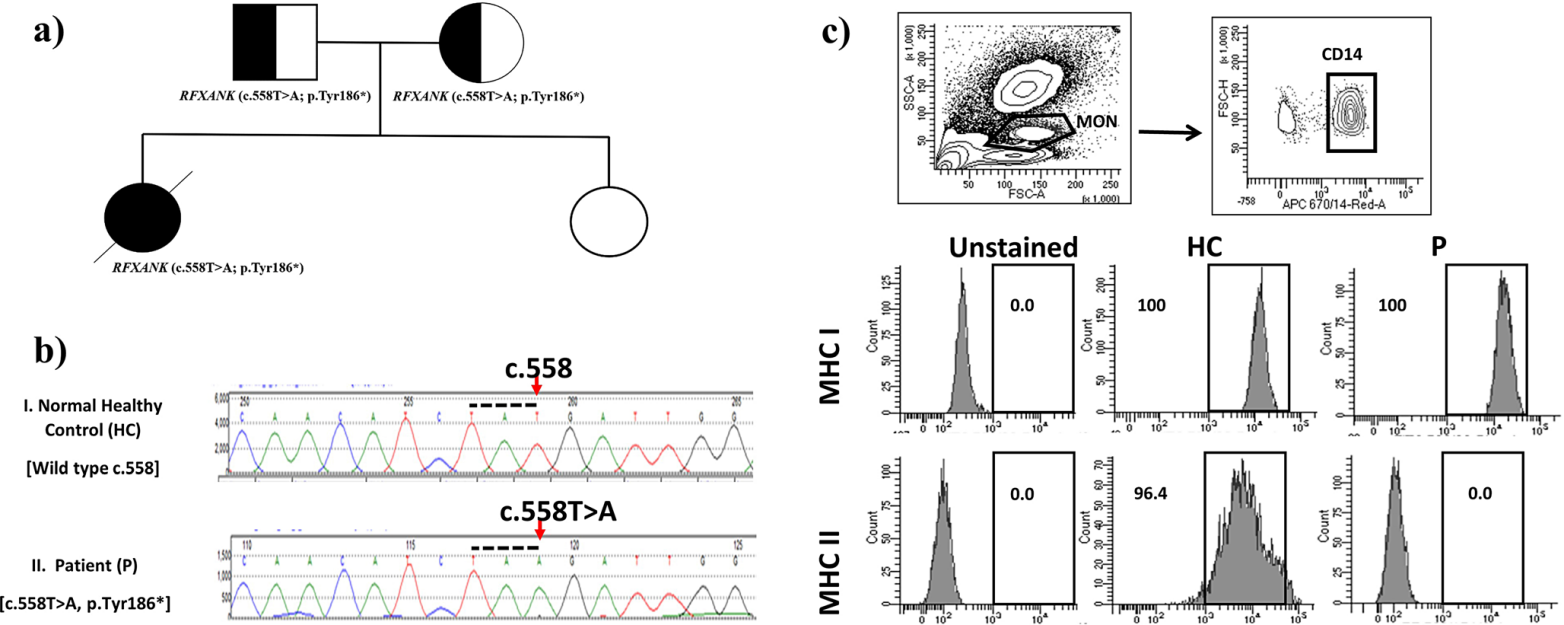
Patient’s pedigree, RFXANK gene DNA sequencing chromatogram, and Major Histocompatibility Complex (MHC) class I and II expression on monocytes. **a**) Pedigree of the family. **b**) RFXANK gene DNA sequencing chromatogram demonstrated the detection of homozygous mutation c.558 A > T, p.Tyr186* in exon7 leading to a premature stope codon in the patient (II) in compare to healthy control (I) with wild nucleotide sequence at c.558. **c**) Flow cytometry gating strategy to identify monocyte (MON) subset from PBMCs and representative histogram of flow cytometry staining for MHC I and MHC II expression for healthy control (HC) and the patient (P). Fluorescence minus one (FMO) was used as negative control (unstained)

A 7-year-old girl, a product of non-consanguineous marriage (Fig. 1a) who was diagnosed at age of 2 years with MHC class II deficiency by genetic and immunological functional studies. Her testing showed a homozygous pathogenic mutation in *RFXANK* (c.558T > A; p.Tyr186*) (Fig. 1b) with an absent HLA-DR expression on monocytes (Fig. 1c). Sanger sequencing was done to the parents and found to be heterozygous carriers of the same mutation. She was managed with monthly IV immunoglobulin and prophylactic antibiotic. Her initial presentation was recurrent sinopulmonary infections and chronic diarrhea. Subsequently, she presented at the age of 4 years with retropharyngeal abscess and generalized lymphadenopathy; her tissue cultures grew *Mycobacterium bovis*, which is probably secondary to the BCG vaccine that she received at birth. She was treated with a one-year course of anti-Tuberculosis (TB) medications with good response then she was kept on two prophylactic anti-TB medications.

**Fig. 2:**
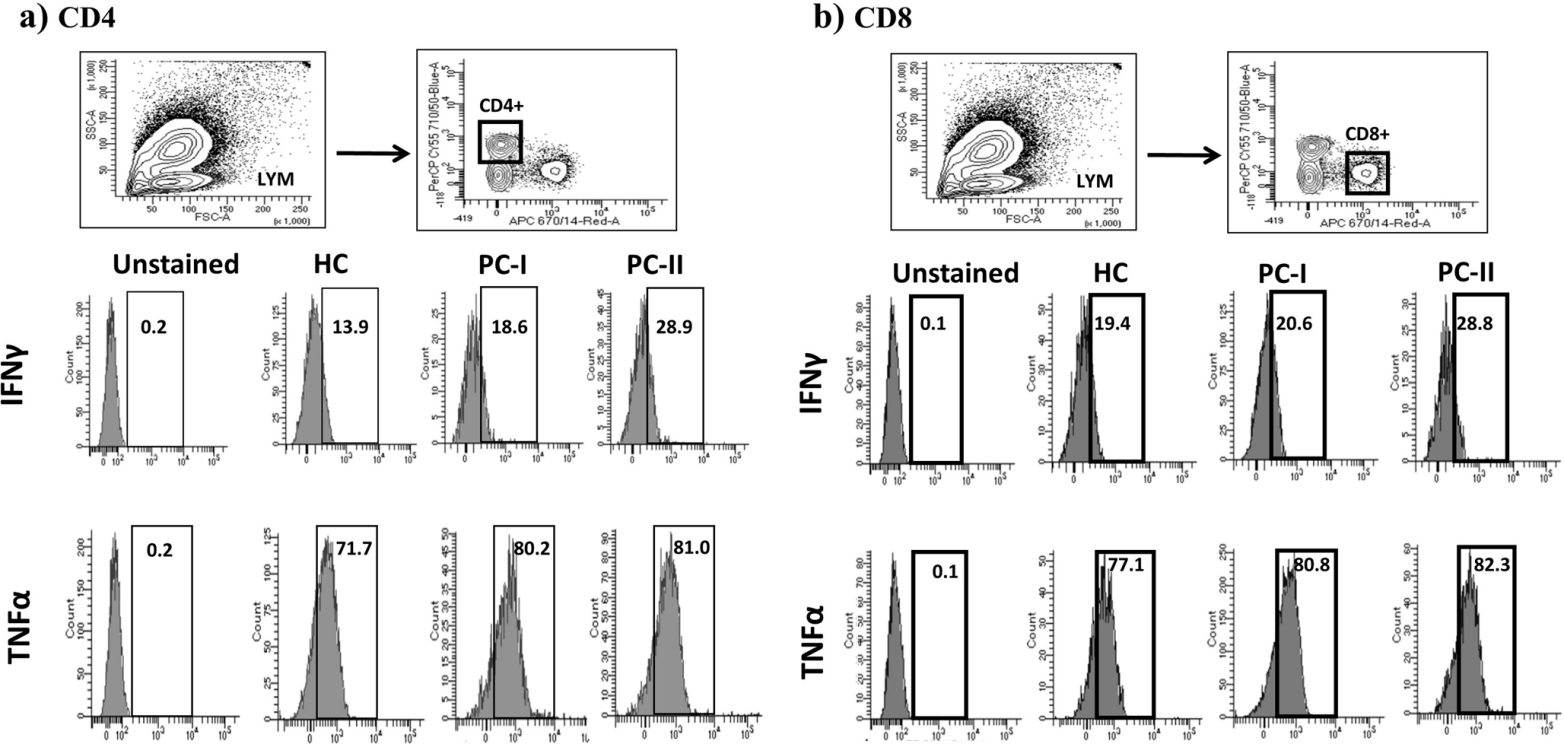
Intracellular expression of Interferon-gamma (IFN‐γ) and tumor necrosis factor alpha (TNF-α). Flow cytometry gating strategy to identify CD4 (**a**) and CD8 (**b**) positive subset from lymphocyte (LYM) and representative histogram of flow cytometry intracellular expression of IFN‐γ and TNF-α for healthy control (HC), positive control I (PC-I) and positive control II (PC-II). Fluorescence minus one (FMO) was used as negative control (unstained)

The presentation with disseminated BCGitis might be attributed to her immune phenotyping that showed severe lymphopenia (Table 1). Repeated lymphocyte subsets post therapy revealed persistent T cell lymphopenia. Previous reports demonstrated that disseminated BCGitis is a very rare presentation in MHCII deficient patients despite receiving BCG vaccination. This protection might be due to the presence of good number of T cells that could secrete protective cytokines like IFN-γ  and TNF-α. To determine whether the T cells from MHC II deficient patient have the ability to produce sufficient amount of those cytokines, we measured the intracellular INFγ and TNFα in two MHC class II deficient patients with a same *RFXANK* genetic mutation and we found that T cells had adequate intracellular inflammatory cytokines (Fig. 2).

**Table 1:** Lymphocyte subsets immunophenotyping with MHC expression by flow cytometry and immunoglobulin levels

Investigations	Patient’s Result	Normal range for age
**Lymphocyte population, cell/mcL**		
Total lymphocyte (cells/mcL)	340	2900–5100 cells/mcL
CD3 cells/mcL	119	1900–3600 cells/mcL
CD3/CD4 cells/mcL	32	600–2000 cells/mcL
CD3/CD8 cells/mcL	70	300–1300 cells/mcL
CD19 cells/mcL	129	300–1200 cells/mcL
CD3-CD16 + CD56 + cells/mcL	76	200–1200 cells/mcL
Total CD25+ %	15	5–10
CD19 + HLADR+ (cells/mcL)	0	12–31
CD3 + CD4 + CD45RA+ %	2	17–40
CD3 + CD4 + CD45RO+ %	11	9–23
CD3 + CD8 + CD45RA+ %	16	15–32
CD3 + CD8 + CD45RO+ %	5	4–15
**Immunoglobulins (Ig), g/L**		
IgA	< 0.01	0.82–4.53 g/L
IgG	2	7.51–15.6 g/L
IgM	0.3	0.46–3.04 g/L
IgE unit/L	< 100	< 100 unti/L
**Vaccine titers**		
Tetanus IgG, IU/mL	N/A*	> 0.15 IU/mL

*Tetanus toxoid Ig was not done as the patient was on monthly immunoglobulin replacement therapy

**Fig. 3:**
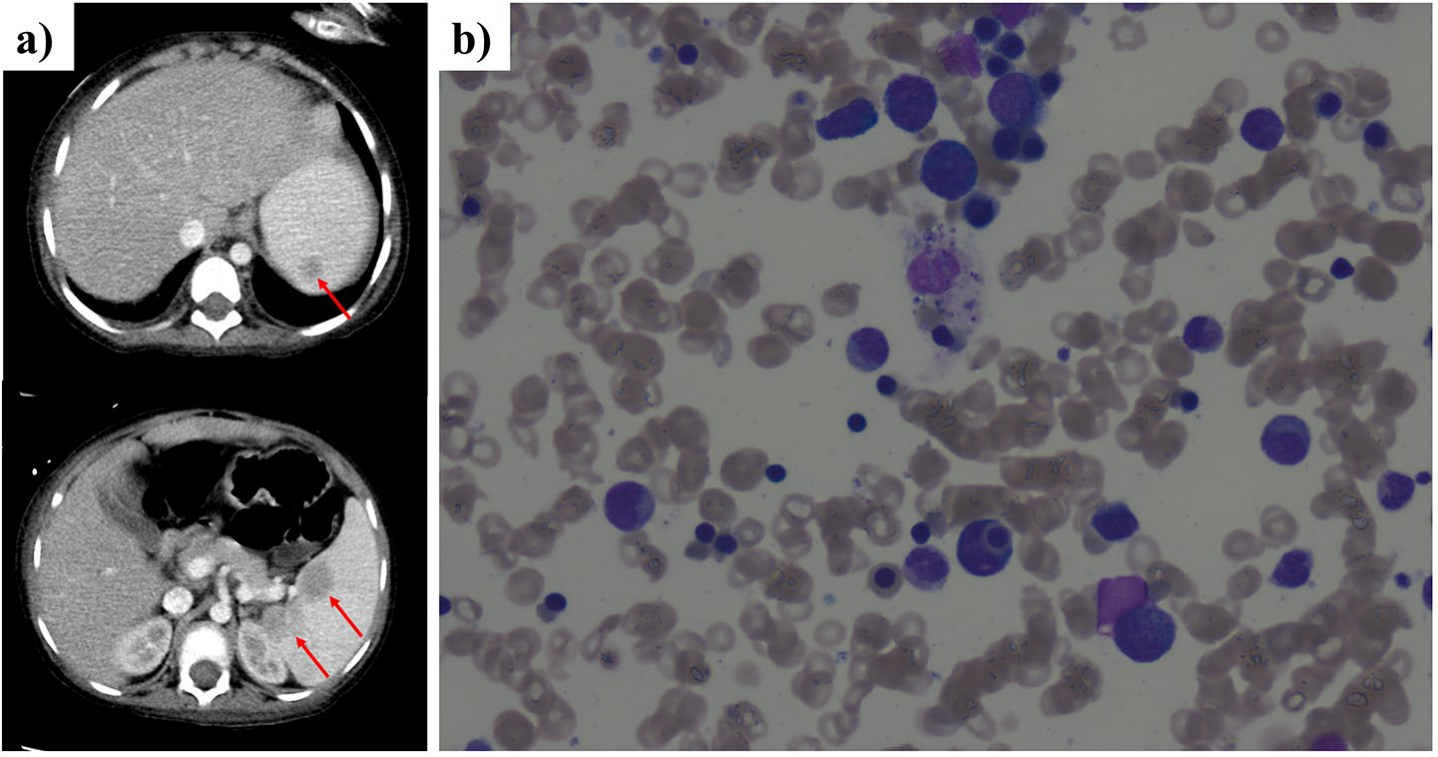
Abdomen CT and bone marrow aspirate findings. (**a**) Abdomen CT scan showed enlarged liver and spleen with multiple abscesses (red arrows). (**b**) BMA showing hemophagocytic activity with macrophages engulfing mainly neutrophils, red blood cells, and platelets. No increased blasts or lymphocytes are observed

At the age of 7 years, she presented to our emergency department with fever, fatigue, and oral mucosal bleeding. Her clinical examination showed high fever [Max. 39.5 C], persistent tachycardia [140-160 s beat/min], ecchymosis and splenomegaly. Her labs revealed pancytopenia, high inflammatory markers, hypofibrinogenemia, hypertriglyceridemia, transaminitis with prolonged coagulation profile and elevated D-dimer, as shown in (Table S1). As this presentation was concerning for sepsis with disseminated intravascular coagulation (DIC), she was started on broad-spectrum antibiotics. Her blood, urine, stool cultures were negative and she continued to be critically ill with worsening laboratory findings including dropping cell lines and prolongation of coagulation profile. Her inflammatory markers showed hyperferritinemia (29,748 ug/L) and high CRP (120) in the setting of decreasing ESR (to 2 mm/hour). Abdominal CT demonstrated an enlarged spleen (14 cm) with an interval development of multiple ill-defined hypodensities suggesting abscesses formation (Fig. 3a). An extensive mycobacterial screening was done, apart from the deferred splenic biopsy due to the high risk of bleeding, with no positive results (Table S2). Her antimicrobial therapies were upgraded to include therapeutic anti-fungal and anti-TB medications. Bone marrow aspiration (BMA) and biopsy showed increased histiocytes with hemophagocytic activity engulfing neutrophils, red blood cells, and platelets (Fig. 3b). No reported evidence of infections or hematologic malignancy. Accordingly, whole exome sequencing looking for HLH-associated genes was sent and came back negative (Supplementary data).

Given the patient’s clinical picture and the laboratory results, the diagnosis of HLH was confirmed as she fulfilled 6 out of 8 HLH diagnostic criteria (Table 2). The patient was started on IV Dexamethasone (10 mg/m^2^), Anakinra (10 mg/kg/day) and IVIG (0.5 mg/kg). After starting the treatment, patient started to show clinical and biochemical evidence of improvement as she became afebrile, she had significant drop in her inflammatory markers and improvement of her cytopenia and transaminitis (Table S1). Subsequently, patient was discharged in good condition with a close follow up and weaning plan of steroid and anakinra. Few months later, she presented to another hospital with fever, shortness of breath, ecchymosis and hemoptysis. Her labs revealed high ferritin, coagulopathy and cytopenia, while her imaging demonstrated a new bilateral chest infiltrate concerning for pneumonia that was complicated by DIC/HLH. She was admitted to PICU for respiratory support and her bronchoalveolar lavage was negative for active mycobacterial infection. During her stay, she developed pulmonary hemorrhage leading to cardiopulmonary arrest and death.

**Table 2:** HLH-2004 diagnostic criteria in the reported case

The diagnosis HLH can be established if one of either 1 or 2 below is fulfilled:	Fulfilled	No	Not done
(1) A molecular diagnosis consistent with HLH		✓	
(2) Diagnostic criteria for HLH fulfilled (five out of the eight criteria below)	✓		
Fever	1/8		
Splenomegaly	2/8		
Cytopenias (affecting ≥ 2 of 3 lineages in the peripheral blood) Hemoglobin < 90 g/L (in infants < 4 weeks: hemoglobin < 100 g/L) Platelets < 100_109/L Neutrophils < 1.0_109/L	3/8		
Hypertriglyceridemia and/or hypofibrinogenemia:	4/8		
Fasting triglycerides ≥ 3.0 mmol/L (i.e., ≥ 265 mg/dl)			
Fibrinogen ≤ 1.5 g/L			
Hemophagocytosis in bone marrow or spleen or lymph nodes. No evidence of malignancy.	5/8		
Low or absent NK-cell activity (according to local laboratory reference)			✓
Ferritin ≥ 500 mg/L	6/8		
Soluble CD25 (i.e., soluble IL-2 receptor) ≥ 2,400 U/ml			✓

HLH: Hemophagocytic lymphohistiocytosis

## Discussion

MHC class II deficiency is a combined immunodeficiency disorder reported mostly in the North Africa and Middle East regions due to high consanguinity rate [2, 3]. This inherited disorder caused by pathogenic defects in any of the four genes *(CIITA, RFXANK, RFX5*, and *RFXAP)* that encode for the regulatory proteins responsible for MHC class II expression [2]. Usually, those patients present with recurrent and severe viral, bacterial, fungal, and protozoal infections during their first years of life [4, 6]. Mostly, they have recurrent sinopulmonary infections, oral thrush and chronic diarrhea with severe malabsorption leading to failure to thrive. Other manifestations include neurological complications and autoimmune cytopenia [4, 8].

Despite the genuine T cell defect in MHC II deficiency due to the lack of antigen presentation, the majority of patients with this immune defect did not suffer from mycobacterial infections even after BCG vaccination. Previous reports demonstrated that the risk of developing disseminated BCGitis in this population is extremely low [3, 4]. We think this protection might be due to the presence of sufficient number of T cells including both T helper and Cytotoxic T cells as the majority of unaffected reported patients had mild to moderate T cell deficiency (> 250 /uL) [3, 4]. Those T cells provide good protective measures that might be attributed to their ability to produce cytokines like IFN‐γ and TNF-α. We measured the intracellular IFN‐γ and TNF-α in two patients with *RFXANK* genetic defect and we found that both CD4 and CD8 cells produce an appropriate amount of those cytokines. For that reason, we thought the disseminated BCGitis in our case is related to the profound T cell lymphopenia. This conclusion was supported by a recent study demonstrating a clear inverse association between the T cell counts and the dissemination of mycobacterial infections [15]. Disseminated BCGitis was reported in two MHC class II deficient patients who had severely reduced CD4 cell counts [7, 8], and our case will be the third one with the same findings. This notion might point to the major role of CD4 population in protection from mycobacterial infections as it might be considered as an important source of inflammatory cytokines, like IFN‐γ and TNF-α [16].

Interestingly, our patient presented at the age of 7 years with a novel clinical and laboratory finding suggesting the diagnosis of HLH. Additionally, an extensive viral, bacterial, mycobacterial and fungal screening did not identify any infectious pathogens. Unfortunately, she had splenic abscesses that were not biopsied due to the high risk of bleeding. Subsequently, the primary-inherited HLH was investigated and genetic testing including next generation sequencing and deletion duplication did not show any HLH disease causing mutations. This presentation expands the clinical manifestation of this combined immunodeficiency disorder.

The pathogenesis of secondary HLH in inborn error of immunity is not fully understood as compared to the primary HLH. Previous reports attributed the risk for HLH, in these inherited disorders, to either a dysregulated immune system leading to uncontrolled release of cytokines or to the presence of persistent infections due to defective protective immunity [10, 13, 17]. In our patient, we think her HLH was triggered by the mycobacterial infection due to her previous history of BCGitis and she was severely lymphopenic, which predisposed her to persistent intracellular organisms. The fact that her infectious screening was negative does not rule out the reactivation of her latent mycobacterial infection considering the presence of splenic abscess that were not cultured, and the long duration of prophylactic anti-TB medications that may inhibit the culture but not enough to eradicate the infection.

Though this report documents rare and novel results related to the mycobacterial susceptibility and the hyperinflammatory phenotype in MHC class II deficiency, it has several limitations. First, some limitations are inherent in its design preventing the generalization and establishing cause and effect relationship. Second, the patient’s splenic lesions were not biopsied, due to the risk of bleeding that renders her infectious work up for HLH triggers inconclusive.

In conclusion, the risk of developing disseminated BCGitis in MHCII deficiency is extremely low and our case will be the third reported case. The increased risk for disseminated BCGitis might be attributed to the severe T helper cell lymphopenia that was documented in our patients and previous reports. Additionally, MHC class II deficiency might be added to the list of inborn errors of immunity that can be complicated with HLH. Therefore, HLH should be considered in any patient with immunodeficiency with limited pathogen control.

### Electronic Supplementary Material

Below is the link to the electronic supplementary material.

Supplementary Material 1

## Data Availability

The original contributions presented in the study are included in the article/Supplementary Materials, further inquiries can be directed to the corresponding author/s.
