# Supplementary material for: Novel Presentation of Major Histocompatibility Complex Class II Deficiency with Hemophagocytic Lymphohistiocytosis
Source: J Clin Immunol. 2024 Mar 1;44(3):73. doi: 10.1007/s10875-024-01674-0 (PMC10904449; doi:10.1007/s10875-024-01674-0)
Supplement: Supplementary file 1 — Supplementary Material 1 [file 10875_2024_1674_MOESM1_ESM.pdf]

Supplementary Data

Supplementary Data: Summary of focused genes list associated with primary immunodeficiency/HLH.

ACD, ACP5, ACTB, ADA, ADAM17, ADAR, AICDA, AIRE, AK2, AP3B1, AP3D1, ARPC1B, ATM, BACH2, BCL10, BCL11B, BLM, BLNK, BLOC1S6, BTK, C1QA, C1QB, C1QC, C1S, C2, C3, CARD11, CARD14, CARD9, CASP10, CASP8, CD19, CD247, CD27, CD3D, CD3E, CD3G, CD40, CD40LG, CD46, CD55, CD59, CD70, CD79A, CD79B, CD81, CD8A, CDC42, CDCA7, CEBPE, CECR1, CFB, CFD, CFH, CFI, CFP, CFTR, CHD7, CIITA, CLCN7, CLPB, COLEC11, COPA, CORO1A, CR2, CSF2RA, CSF2RB, CSF3R, CTC1, CTLA4, CTPS1, CTSC, CXCR4, CYBA, CYBB, DCLRE1C, DDX58, DGKE, DKC1, DNAJC21, DNMT3B, DOCK2, DOCK8, ELANE, EPG5, ERCC6L2, EXTL3, FAAP24, FADD, FAS, FASLG, FERMT3, FOXN1, FOXP3, G6PC3, G6PD, GATA2, GFI1, GINS1, AP3D1, HAX1, HELLS, HYOU1, ICOS, IFIH1, IFNAR2, IFNGR1, IFNGR2, IGLL1, IKBKB, IKZF1, IL10, IL10RA, IL10RB, IL12B, IL12RB1, IL17RA, IL17RC, IL1RN, IL21, IL21R, IL2RA, IL2RG, IL36RN, IL7R, IRAK4, IRF2BP2, IRF8, ISG15, ITGB2, ITK, JAGN1, JAK1, JAK3, KRAS, LAMP1, LAMTOR2, LAT, LCK, LIG4, , LIPA, LPIN2, LRBA, LYST, MAGT1, MALT1, MAP3K14, MASP1, MEFV, MKL1, MOGS, MRE11A, MSN, MTHFD1, MVK, MYD88, MYO5A, NBN, NCF1, NCF2, NCF4, NCSTN, NCKAPL1,NFKB1, NFKB2, NFKBIA, NHEJ1, NHP2, NLRC4, NLRP1, NLRP12, NLRP3, NOD2, NOP10, NRAS, NSMCE3, OFD1, ORAI1, OTULIN, PARN, PEPD, PGM3, PIGA, PIK3CD, PIK3CG, PIK3R1, PLCG2, PMS2, PNP, POLE, POLE2, PRF1, PRKCD, PRKDC, PSENEN, PSMB8, PSTPIP1, PTPRC, RAB27A, RAC2, RAG1, RAG2, RASGRP1, RBCK1, RECQL4, RFX5, RFXANK, RFXAP, RHOH, RhoG, RLTPR, RMRP, RNASEH2A, RNASEH2B, RNASEH2C, RNF168, RNF31, RNU4ATAC, RORC, RPSA, RTEL1, SAMD9, SAMD9L, SAMHD1, SBDS, SERPING1, SH2D1A, SLC29A3, SLC35C1, SLC37A4, SLC46A1, SLC7A7, SMARCAL1, SMARCD2, SP110, SPINK5, SRP72, STAT1, STAT2, STAT3, STAT5B, STIM1, STK4, STX11, STXBP2, TAP1, TAP2, TAPBP, TBX1, TBXAS1, TCF3, TCN2, TERC, TERT, TFRC, THBD, TINF2, TMC6, TMC8, TMEM173, TNFAIP3, TNFRSF13B, TNFRSF1A, TNFRSF4, TRAF3IP2, TREX1, TRNT1, TTC7A, TYK2, UNC119, UNC13D, UNC93B1, UNG, USB1, USP18, VPS13B, VPS45, WAS, WDR1, WIPF1, WRAP53, XIAP, ZAP70, ZBTB24, ZNF341, ZNFX1.

Table S1. Trend of laboratory results during the presentation.

Investigations	Day 1	Day 2	Day 3	Day 4	Day 5	Day 10	Normal Range (Unit)
**WBC**	1.23	0.5	0.28	0.62	0.87	3.05	4 - 12 (10^9 cells/L)
**Neutrophils**	1.02	0.3	N	0.32	0.64	2.36	1.1 - 7.2 (10^9 cells/L)
**Hemoglobin**	87	74	64	91	80	118	113 – 150 (gm/L)
**Platelet**	26	7	17	24	55	74	150 - 400 (10^9 cells/L)
**CRP**	-	147	-	-	-	3	<3.50 (mg/L)
**ESR**	26	2	-	-	-	4	0–15 (mm/hour)
**Ferritin**	19759	29748	>33511	29052	20530	236.9	4.6 - 204 (ug/L)
**Fibrinogen**	1.71	1.44	1.07	1.39	1.49	1.62	1.5 - 4.1 (gm/L)
**D-dimer**	>35.2	>35.2	>35.2	N	20.4	0.82	0-0.5 (mg/L)
**PTT**	39.1	63.6	36.2	32.1	27.9	26.8	25 - 33
**PT**	13.5	18.1	12.3	12.5	13.2	12	9.4 - 12.3
**INR**	1.24	1.7	1.12	1.14	1.12	1.09	0.8 - 1.2
**GGT**	55.1	65	105.3	120.4	113.6	48.5	9-36 (U/L)
**AST**	93	161	132	98	97	25	5-34 (U/L)
**ALT**	27	22	27	28	39	15	6-54 (U/L)
**Albumin**	35	29	33	36	35	36	39-53 (g/L)
**Triglycerides**	1.33	1.95	1.94	2.72	1.88	0.76	< 1.69 (mmol/L)

WBC, white blood cells; CRP, C-reactive protein; ESR, erythrocyte sedimentation rate; PTT, partial thromboplastin time; PT, prothrombin time; INR, international normalized ratio; GGT, gammaglutamyl transferase; AST, aspartate aminotransferase; ALT, alanine aminotransferase.

Table S2. Summary of infectious screening testing performed during the patient's presentation (all negative).

Blood	NPA	Bronchoalveolar lavage	Lymph node biopsy
Bacterial culture	Viral multiplex PCR	Bacterial stain and culture	Bacterial stain and culture
Fungal culture	**Urine**	Fungal stain and culture	Fungal stain and culture
Tuberculosis IGRA	Bacterial culture	AFB stain and culture	AFB Stain and culture
Cryptococcal antigen	Fungal culture	TB PCR	TB PCR
Aspergillus galactomannan	**Stool**	Aspergillus antigen	
Coccidioides antibodies	Bacterial culture	PCP direct fluorescent antigen	
Blastomyces antibodies	Ova and parasite	**Bone marrow aspirate**	
Herpes Simplex Virus PCR	C. difficile toxins	Bacterial culture	
Adenovirus PCR	Cryptosporidium	Fungal culture	
EBV PCR		AFB stain and culture	
CMV PCR		TB PCR	

IGRA, Interferon-gamma release assay; PCR, Polymerase chain reaction; EBV, Epstein-Barr virus; CMV, Cytomegalovirus; C. difficile, Clostridioides difficile; AFB, Acid fast bacillus; TB, tuberculosis.
